# Clinical and Genetic Insights Into Isolated Proteinuria With *CUBN* Variants

**DOI:** 10.1016/j.ekir.2025.103754

**Published:** 2025-12-29

**Authors:** Nana Sakakibara, Shinya Ishiko, Yu Tanaka, Yuta Inoki, Yuta Ichikawa, Hideaki Kitakado, Chika Ueda, Atsushi Kondo, Yuya Aoto, Tomoko Horinouchi, Tomohiko Yamamura, Shingo Ishimori, Chinatsu Onodera, Aya Inaba, Riku Hamada, Yutaka Harita, China Nagano, Kandai Nozu

**Affiliations:** 1 Department of Pediatrics, Kobe University Graduate School of Medicine, Kobe, Japan; 2 Department of Pediatrics, School of Medicine, Iwate Medical University, Morioka, Japan; 3 Department of Pediatrics, Yokohama City University Medical Center, Yokohama, Japan; 4 Department of Nephrology, Tokyo Metropolitan Children’s Medical Center, Tokyo, Japan; 5 Department of Pediatrics, Graduate School of Medicine, The University of Tokyo, Tokyo, Japan

**Keywords:** chronic benign proteinuria, Cubilin, *CUBN*, Imerslund–Gräsbeck syndrome

## Abstract

**Introduction:**

Cubilin is a multiligand receptor essential for vitamin B12 uptake in the small intestine and for low–molecular-weight protein reabsorption in the proximal tubule. Biallelic *CUBN* variants cause Imerslund–Gräsbeck syndrome (IGS), often accompanied by proteinuria, whereas C-terminal variants have been associated with autosomal recessive chronic benign proteinuria. This study aimed to clarify the genetic and clinical characteristics of chronic benign proteinuria and the molecular basis distinguishing it from IGS.

**Methods:**

We evaluated patients with proteinuria carrying biallelic *CUBN* variants identified through targeted panel sequencing and investigated molecular mechanisms underlying the phenotypic differences between IGS and chronic benign proteinuria.

**Results:**

Fifty-two patients from 42 families were analyzed, and 40 *CUBN* variants, including 30 novel variants, were identified. All patients presented with incidentally detected subclinical proteinuria—most commonly during the 3-year-old mass urinary screening—and none showed hypoalbuminemia, impaired kidney function, or response to renin-angiotensin system (RAS) inhibitors. Light microscopy revealed minor glomerular abnormalities, whereas electron microscopy frequently demonstrated thin basement membranes or mild foot process effacement. We identified a novel *CUBN* transcript containing an open reading frame that produces a truncated protein with a unique C-terminus. Full-length cubilin was expressed only in the kidney, whereas a smaller isoform was present in both kidney and small intestine.

**Conclusion:**

Patients with biallelic *CUBN* variants exhibit preserved kidney function despite subtle glomerular changes. Because only the truncated cubilin isoform is expressed in the intestine, C-terminal variants do not affect vitamin B12 absorption, thereby explaining the absence of malabsorption in chronic benign proteinuria associated with *CUBN* C-terminal variants.

## Graphical abstract

**Figure:**
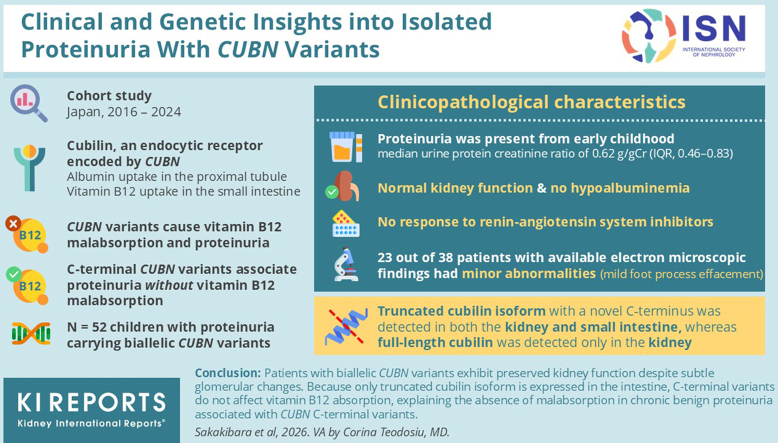

In healthy individuals with normal kidney function, the amount of albumin excreted in the urine is typically < 30 mg/d.^1^ This minimal excretion is due to the selective filtration of small-molecular-weight proteins in the glomerulus and reabsorption in the proximal tubules. Under normal physiological conditions, the amount of albumin that passes through the glomerulus each day is estimated to be several grams. However, albumin is rarely detectable in the urine because of its endocytosis by cubilin, which is expressed in the proximal tubule.^2^^,^^3^

Cubilin is encoded by *CUBN*, which is a large, 460 kDa, endocytic receptor that mediates the uptake of the intrinsic factor vitamin B12 complex in the small intestine and the uptake of filtered proteins, such as albumin, in the kidney proximal tubule.^4^ The structure of *CUBN* consists of an N-terminal region encompassing a coiled-coil region followed by 8 epidermal growth factor–like domains and 27 complement C1r/C1s, Uegf, and Bmp1 (CUB) domains.^5^ The CUB domain cluster harbors distinct sites for ligand binding, and the binding site for intrinsic factor vitamin B12 is localized in the CUB 5 to 8 domains (CUB5–8).^6^

*CUBN* is the causative gene for autosomal recessive vitamin B12 malabsorption syndrome, IGS,^7^ which often presents with proteinuria. Variants of *CUBN* have been detected in patients presenting with asymptomatic proteinuria (albuminuria) only.^8^^,^^9^ Furthermore, in a French cohort of 759 patients with suspected genetic kidney disease, 39 with biallelic variants in *CUBN* located downstream of the vitamin B12 binding site exhibited proteinuria without vitamin B12 malabsorption syndrome and normal kidney function.^10^ This was a surprising finding because albuminuria is a well-known risk factor for chronic kidney disease and end-stage kidney disease.^11^, ^12^, ^13^ In addition, patients with *CUBN* variants presenting with asymptomatic proteinuria are characterized by moderate-to-severe proteinuria since childhood, no pathological changes, and a lack of response to RAS inhibitors, but no kidney dysfunction. This rare condition has attracted attention as a new disease concept, known as chronic benign proteinuria.^14^

Previous genetic studies show that although all IGS variants are located before or within CUB8,^4^^,^^15^ chronic benign proteinuria is caused by variants located posterior to CUB8.^10^^,^^14^ Overall, these results led us to hypothesize that the cubilin isoform lacking the C-terminus after CUB8 is mainly expressed in the small intestine. In other words, if the variant is located posterior to CUB8, this shorter isoform may not be damaged and thus does not cause vitamin B12 malabsorption. However, full-length cubilin might be damaged, thus causing albumin malabsorption. Bedin *et al.*^10^ reported the presence of a presumed intestinal transcript immediately truncated after CUB8, referencing the Genotype-Tissue Expression database,^16^ and reported that premature truncation of cubilin is likely to occur after CUB8 in normal populations from the in-house genome database and gnomAD.^10^ However, it is not clear what the truncated *CUBN* transcript is, and whether the protein is actually translated from this intestinal transcript. Therefore, in an attempt to understand why C-terminal variants do not cause vitamin B12 malabsorption symptoms, in this study, we aimed to provide genetic and clinical information on chronic benign proteinuria and verify the phenotypic differences between IGS and chronic benign proteinuria.

## Methods

### Patients

From January of 2016 to December of 2024, we performed targeted panel sequencing for 892 patients with proteinuria, including congenital nephrotic syndrome, infantile nephrotic syndrome, steroid-resistant nephrotic syndrome, and asymptomatic proteinuria found in a chance urinalysis. We collected the clinical findings and laboratory data of patients harboring biallelic *CUBN* variants. Data were collected from questionnaires submitted by local doctors at the time of genetic testing. Laboratory data were obtained at the time of genetic testing.

Laboratory data are shown as the median and interquartile range (IQR). Statistical analyses were performed using Microsoft Excel 2019 (Microsoft Corporation, Redmond, WA).

### Genetic Testing

Genomic DNA was extracted from peripheral blood leukocytes of patients using QuickGene DNA Whole Blood Kit S (Kurabo Industrial Ltd., Osaka, Japan) or QuickGene-Auto S DNA Blood Kit (Kurabo Industrial Ltd.), in accordance with the manufacturer’s instructions. A comprehensive analysis was conducted on the genes responsible for inherited kidney diseases using targeted next-generation sequencing according to the manufacturer’s instructions. HaloPlex HS and SureSelect target enrichment system kits (Agilent Technologies, Santa Clara, CA) are customizable panel kits used to sequence genes. A list of the genes screened using targeted sequencing is shown in Supplementary Table S1.

All indexed DNA samples were amplified using polymerase chain reaction (PCR) and sequenced on a MiSeq platform (Illumina, San Diego, CA). Data from alignment to mutation categorization were analyzed using SureCall software (Agilent Technologies). As previously reported,^17^ cases with suspected copy number variations containing *CUBN* in the application-based pair analysis were subjected to custom array–based comparative genomic hybridization using Cytogenomics software (Agilent Technologies). The variants detected by next-generation sequencing were confirmed by direct Sanger sequencing using a 3130 Genetic Analyzer or SeqStudio Genetic Analyzer (Thermo Fisher Scientific, Tokyo, Japan). The *CUBN* cDNA reference number is NM_001081.4 (NCBI for Biotechnology Information ID).

Pathogenicity was predicted in accordance with the American College of Medical Genetics guidelines. To prioritize candidate novel exonic variants, SIFT (https://sift.bil.astar.edu.sg/), PolyPhen-2 (https://genetics.bwh.harvard.edu/pph2/), Mutation Taster (https://www.mutationtaster.org/), and CADD (https://cadd.gs.washington.edu/snv) were used. Splice site variants of uncertain pathogenicity were evaluated by splicing pattern analysis using a minigene assay, as previously reported.^18^

All procedures involving humans were reviewed and approved by the Institutional Review Board of Kobe University Graduate School of Medicine (approval no. 301) and performed in accordance with the Declaration of Helsinki Declaration of 1964 and its later amendments. Written informed consent was obtained from all the patients and/or their parents before genetic testing.

### Cloning of the Novel Transcript

To clone a novel transcript variant of *CUBN*, rapid amplification of cDNA 3’ ends (i.e., 3'-RACE) was performed. PCR was carried out using a human small intestine cDNA library (Human Small Intestine Marathon-Ready cDNA, Takara Bio) as a template. PCR amplification was conducted first with AP1 (supplied by the kit: 5’-CCATCCTAATACGACTCACTATAGGGC-3’) and GSP1 (*CUBN* exon 20-21 boundary: 5’–CAGTGCTGAGGATTTGGCATGTG–3’). Nested PCR was then carried out using AP2 (supplied by the kit: 5’-ACTCACTATAGGGCTCGAGCGGC-3’) and GSP2 (*CUBN* exon 27 - 28 boundary: 5’–AGCTCTATGATGGACCACGG–3’) as PCR primers according to the manufacturer’s instructions. The Advantage 2 Polymerase Mix (Takara Bio) was used for PCR analysis. PCR-amplified products were gel-purified and sequenced directly with GSP2 as a sequencing primer. Isoform sequencing (Iso-Seq), a long-read RNA sequencing method that enables identification of the transcriptome, was performed using human kidney total RNA and human ileum total RNA. The data obtained from Iso-Seq were used to guide the design of the 3' RACE experiments. Detailed methods and data are provided in Supplementary Figure S1, including its accompanying legend.

### Immunofluorescence

Double-immunofluorescence staining of cubilin and CD10 was performed using paraffin-embedded sections of 3-μm thickness of normal human kidney and human small intestine specimens (BioChain), and kidney biopsy tissue of Neph 224. CD10 was used as a marker for the proximal tubules. After incubation at 60 °C for 10 minutes, the sections were deparaffinized by 2 changes of xylene and washed with 95%, 90%, 80%, and 70% concentrations of ethanol rinses, and subsequently washed in distilled water. Antigen retrieval was performed using the wet autoclaving method by placing the tissue sections in citrate buffer (pH: 9.0). To block endogenous peroxidases, the sections were incubated with hydrogen peroxide 3% (Dako Japan, Tokyo, Japan) for 5 minutes at room temperature. After washing several times with phosphate-buffered saline, the sections were blocked with 10% goat serum at room temperature for 30 minutes. Following phosphate-buffered saline washing several times, the sections were incubated overnight in either cubilin and CD10 primary antibody prepared at appropriate dilution in phosphate-buffered saline in a humidified chamber at 4 °C. After washing with phosphate-buffered saline, the sections were stained with Alexa Fluor conjugated secondary antibodies at 37 °C for 30 minutes. Details of the primary and secondary antibodies used in this study are presented in Supplementary Table S2. The stained sections were viewed under a fluorescence microscope (BZ-9000 and BZ-X700; KEYENCE, Osaka, Japan).

### Western Blot

For conventional western blot analysis, human kidney whole tissue lysate (#NB820–59231, Novus Biologicals, Littleton, CO) and human small intestine ileum whole tissue lysate (#NB820-59256, Novus Biologicals, Littleton, CO) were solubilized in Laemmli sample buffer (Bio-Rad Laboratories, Hercules, CA). Equal amounts of protein (25 μg per lane) were resolved on a 4% to 20% gradient sodium dodecyl sulphate-polyacrylamide gel electrophoresis and transferred to a polyvinylidene fluoride membrane (Bio-Rad Laboratories). After blocking for 1 hour at room temperature, cubilin and β-actin antibodies were used for primary antibody incubation performed at 4 °C overnight. Samples were then incubated with horseradish peroxidase-conjugated secondary antibodies at room temperature for 1 hour. The proteins were visualized using a ChemiDoc MP imaging system (Bio-Rad Laboratories).

Automated capillary western analysis (Abby Simple Western automated western blots system; ProteinSimple) with 66 to 440 kDa separation module was also performed to analyze the human kidney whole tissue lysate and human small intestine ileum whole tissue lysate following the manufacturer’s instructions. Data were analyzed using the Compass for Simple Western software version 6.0.0 (Protein Simple) to automatically calculate the molecular weight of the detected proteins. Detailed information on the primary and secondary antibodies are provided in Supplementary Table S2.

## Results

### Variants Detected

**Figure 1:**
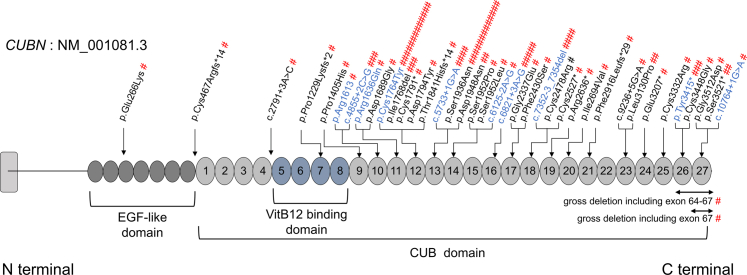
*CUBN* variants detected in this study along the cubilin protein. Novel variants are shown in black, and the reported variants are shown in blue. #Number of families detected. We detected 40 types of *CUBN* variants, 30 of which were novel. Eleven variants were detected in multiple families, and p.Cys1764Tyr and p.Ser1936Asn were detected in the largest number of 13 families.

We identified 52 patients with biallelic pathogenic CUBN variants in the *CUBN*. We detected 40 types of *CUBN* variants, 30 of which were novel, and 3 of which we have already reported.^19^^,^^20^ In Figure 1 and Supplementary Table S3, we present details of the variants detected in this study. Segregation information of *CUBN* variants is shown in Supplementary Table S4. Eleven variants were detected in multiple families, among which p.Cys1764Tyr and p.Ser1936Asn were detected in the largest number of 13 Japanese families. The 3 noncanonical intronic variants (c.2791+3A>C, c.6821+3A>G, and c.9236+5G>A) were evaluated for splicing using the minigene assay and were found to be aberrant in splicing (Supplementary Figure S2).

### Clinicopathological Characteristics

The clinicopathological characteristics of the patients are presented in Table 1. The median age at first detection of proteinuria was 3 (IQR: 3–3) years. All patients were incidentally found to have proteinuria on urinalysis screening. Most patients were noted to have proteinuria on Japanese unique mass urinalysis for all 3-year-old children living in Japan. The median age at genetic diagnosis was 7 (IQR, 4–12) years. At the age of genetic testing, there were no cases with hypoalbuminemia, the median urine protein-to-creatinine ratio level was 0.62 (IQR: 0.46–0.83) g/gCr, the median urine β_2_-microglobulin was 180 (IQR: 112–248) μg/l, and the median Cr-eGFR level was 112.0 ml/min per 1.73 m^2^ (IQR: 105.5–121.1). All patients had normal kidney function except 1 (Neph496) with a Cr-eGFR of 81 ml/min per 1.73 m^2^ at the time of genetic testing, which we confirmed to have normalized kidney function after stopping the RAS inhibitor. Thirty-five patients received RAS inhibitor treatment but showed no response.

**Table 1:** Clinical characteristics of patients with *CUBN* variants

No.	Age at genetic diagnosis (yr)	Age at first detection of proteinuria (yr)	Gender	Cr-eGFR (ml/min per 1.73 m^2^)	S-Alb (g/dL)	uPro/Cr (g/gCr)	uβ2MG (μg/l)	Pathology (light microscopy)	Pathology (electron microscopy)	Treatment	Type of screening urinalysis
A729	10	3	F	134.8	4.4	0.59	ND	1st: MGA 2nd: FSGS	TBM	ARB, ACE-I	3-yr-old
Neph17	10	3	F	140.8	4.6	0.59	ND	MGA	Unremarkable	ARB	Preschool
Neph195	4	3	F	121.8	4.3	0.83	LOD	MGA	No Data	ACE-I	3-yr-old
Neph224	3	3	M	112	5	1.1	208	MGA	FPE partial	ARB	3-yr-old
Neph255	4	2	F	110.5	4.8	1.11	95	MGA	Unremarkable	ACE-I	Preschool
Neph278	6	3	M	110.11	4.4	0.99	97	MGA	FPE	ARB	Preschool
Neph358	4	1	M	115.8	4.9	1.4	762	MGA	Unremarkable	ACE-I	Post-streptococcal infection
Neph359	6	2	M	105.8	4.2	0.83	148	MGA	FPE partial TBM partial	ARB	Preschool
Neph423	18	3	M	123.1	4.9	0.32	293	MGA	Unremarkable	Untreated	3-yr-old
Neph496	7	3	M	81→99.1	4	0.4	100	MGA	FPE	ACE-I, ARB	3-yr-old
Neph496 brother	5	3	M	101	4.2	0.46	100	MGA	FPE partial	ACE-I	3-yr-old
Neph515	10	6	F	104.6	4.6	0.56	ND	MGA	Unremarkable	ACE-I	Elementary school
Neph527	9	4	M	114.29	4.2	0.37	330	MGA	Unremarkable	ACE-I	Medical check-up
Neph527 brother	4	4	M	101.4	4	0.34	427	Not Done	Not Done	ACE-I	Preschool
Neph558	3	3	F	110.5	4.5	1.54	97	Mesangial proliferation	TBM	Untreated	3-yr-old
Neph558 brother	3	3	M	108.06	4.7	1.14	119	Mesangial proliferation	Unremarkable	No Data	3-yr-old
Neph576	20	3	M	105	5	0.78	244	MGA	Unremarkable	ARB	3-yr-old
Neph576 sister 1	20	3	F	93.1	4.4	0.69	98	Not Done	Not Done	ARB	3-yr-old
Neph576 sister 2	20	3	F	100.3	4.4	0.61	151	MGA	Unremarkable	ARB	3-yr-old
Neph580	10	4	F	109	4.7	0.77	LOD	MGA	TBM	ACE-I	Preschool
Neph603	11	3	F	113.13	4.2	0.56	LOD	MGA	TBM	ARB	3-yr-old
Neph606	4	3	M	107.28	4.5	0.81	115	MGA	FPE	ACE-I, ARB	Preschool
Neph624	7	5	M	138.7	4	0.29	214	Not Done	Not Done	ARB	Performed by chance
Neph627	9	1	M	112.75	4.6	0.62	207	MGA	Unremarkable	ACE-I	Preschool
Neph627 sister	2	1	F	101.98	4.6	1.42	481	Not Done	Not Done	Untreated	Preschool
Neph635	12	9	M	96.6	4.5	0.73	53	MGA	Unremarkable	No Data	Elementary school
Neph642	5	3	M	127.03	4.5	1.1	118	MGA	FPE	ACE-I	3-yr-old
Neph655	17	?	M	104	4.8	0.3	430	Not Done	Not Done	Untreated	Preschool
Neph658	3	2	M	142.35	4.4	0.41	ND	MGA	FPE TBM	ARB	Preschool
Neph668	5	3	M	101	4.3	0.54	273	MGA	FPE	ACE+ARB	3-yr-old
Neph697	6	3	M	98.01	4.4	0.38	158	MGA	FPE partial, TBM partial	ARB	3-yr-old
Neph697 sister	8	3	F	123.84	4.6	0.79	98	MGA	FPE partial	multi immunosuppressants	3-yr-old
Neph708	10	3	M	107.92	4.7	0.86	155	MGA	No Data	ACE-I	3-yr-old
Neph711	16	3	F	126.8	4.1	0.46	ND	MGA	No Data	ACE+ARB	3-yr-old
Neph716	13	3	M	115.11	4.5	0.7	118	MGA	FPE partial	ARB	3-yr-old
Neph719	16	6	M	111.95	4.5	0.76	130.9	Not Done	Not Done	Untreated	Elementary school
Neph719 brother 1	12	4	M	132.04	4.4	0.59	187.5	Not Done	Not Done	Untreated	Post-streptococcal infection
Neph719 brother 2	7	3	M	107.69	4.5	0.51	200.7	MGA	FPE TBM	Untreated	3-yr-old
Neph732	7	3	F	105.6	5.3	0.53	107	MGA	FPE partial	ARB	3-yr-old
Neph732 sister	5	3	F	117.7	4.6	0.85	224	MGA	FPE partial	ARB	3-yr-old
Neph749	4	3	F	108.8	4.7	1.12	180	Not Done	Not Done	Untreated	3-yr-old
Neph761	12	2	M	113.9	4.4	0.24	154	MGA	Unremarkable	Untreated	Preschool
Neph766	13	3	M	139.1	4.4	0.4	730	MGA	No Data	Untreated	Preschool
Neph771	4	3	F	120.9	4.5	0.71	186	MGA	Unremarkable	ACE-I	3-yr-old
Neph781	7	6	M	88.89	4.5	0.62	190	MGA	FPE partial TBM	ACE-I	Elementary school
Neph800	5	3	M	115.19	4.1	0.81	108	MGA	FPE	Untreated	3-yr-old
Neph811	17	3	M	113	4.3	0.62	356	MGA	No Data	Untreated	3-yr-old
Neph815	8	7	M	116.9	4.8	0.56	103	MGA	No Data	Untreated	Elementary school
Neph819	4	3	M	126	4.2	0.4	212	MGA	Unremarkable	Untreated	3-yr-old
Neph845	12	7	F	137.46	4.1	0.44	256	Mesangial proliferation	Unremarkable	ARB	Elementary school
Neph860	12	3	F	119.6	4.3	0.42	ND	MGA	Winkling of basement membrane	ARB	Preschool
Neph865	3	3	M	110.2	4.4	1.07	251	MGA	FPE partial	ARB	3-yr-old

3-year-old, 3-year-old mass urinary screening; ACE-I, angiotensin-converting-enzyme inhibitor; ARB, angiotensin II receptor blocker; elementary school, annual school mass urinary screening; F, female; FPE, foot process effacement; FSGS, focal segmental glomerulosclerosis; LOD, under limit of detection; M, male; MGA, minor glomerular abnormality; poststreptococcal infection, screening urinalysis for detection of poststreptococcal acute glomerulonephritis in patients with streptococcal pharyngitis or tonsillitis; preschool, annual urinalysis at kindergarten and nursery; S-Alb, serum-albumin; TBM: thin basement membrane; uPro/Cr, urine protein creatinine ratio; uβ2MG, urine β_2_-microglobulin.

Of the 44 patients who underwent kidney biopsy, 41 had minor glomerular abnormalities and 3 had mild mesangial proliferative nephritis on light microscopy. One patient showed, in the first kidney biopsy, a minor glomerular abnormality; however, the second kidney biopsy showed focal segmental glomerulosclerosis (FSGS). Electron microscopic findings were available for 38 patients, and we observed that 23 of these patients have some of the following electron microscopic abnormalities: thin basement membrane (*n* = 9), wrinkling of the basement membrane (*n* = 1), and/or mild foot process effacement (*n* = 18). Electron microscopy images representing these findings are shown in Supplementary Figure S3.

### Detection of Novel Transcripts

**Figure 2:**
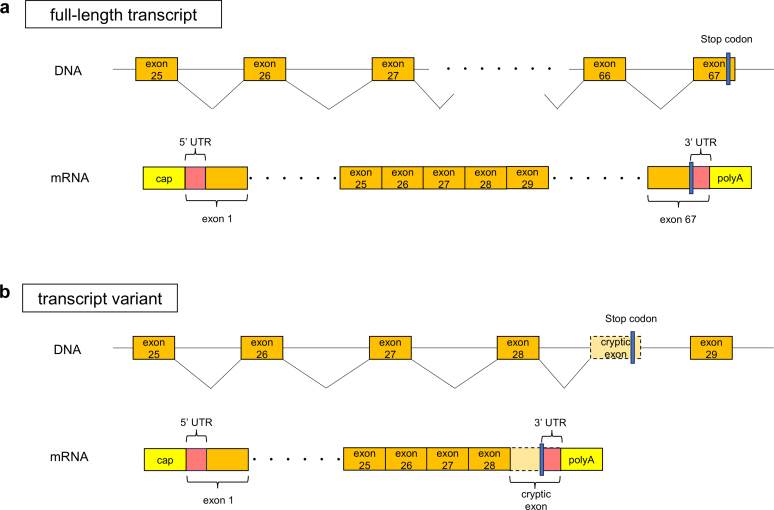
Schematic of *CUBN* transcript variants. (a) Schematic representation of the known full-length *CUBN* transcript. A stop codon was present in the terminal exon (exon 67). (b) Schematic representation of the alternative *CUBN* transcript variants. Alternative splicing generates the cryptic exon in intron 28 as a new terminal exon harboring a stop codon, leading to changes in the 3′-coding and 3′ UTR sequences. This splicing variant may encode a cubilin isoform with a truncated C-terminus.

We successfully cloned the novel transcript, which represents a splicing variant of *CUBN* by the 3'-RACE method using the human small intestine cDNA library. In this transcript, a cryptic exon beginning with g.17,001,178 (c.4169-10663) in intron 28 was generated posterior to exon 28, and a poly(A) tract was added after the polyadenylation signal (AAUAAA) in g.17,000,563-g.17,000,558 (Supplementary Figure S4). This novel transcript variant contained an open reading frame corresponding to a truncated protein with a novel C-terminus containing CUB5–8 domains (Figure 2).

### Evaluation of Protein Expression

**Figure 3:**
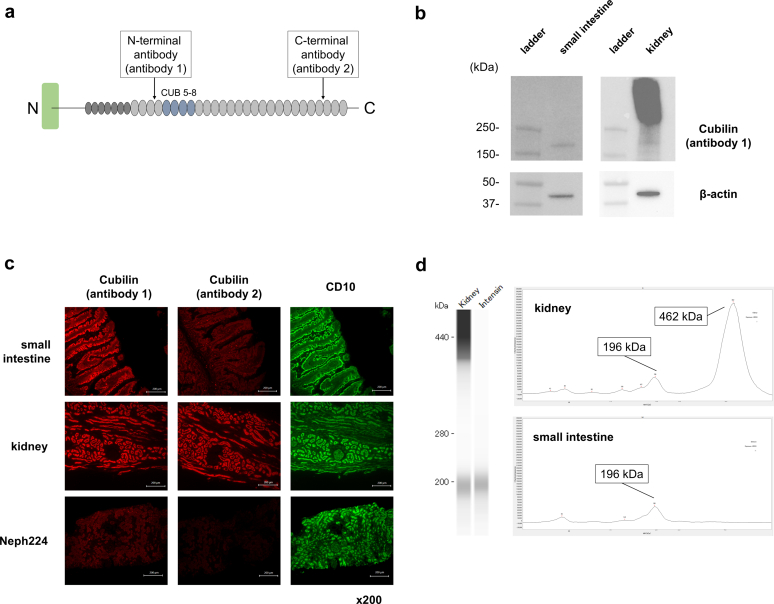
Evaluation of cubilin isoform protein expression by immunofluorescence and western blot of kidney and small intestine tissues. (a) Mapping of the recognition sites of anti-cubilin antibodies. N-terminal antibody (antibody 1) is against the region before CUB5–8, and C-terminal antibody (antibody 2) is against the region posterior to CUB5–8. (b) Immunofluorescence staining of cubilin (red) and CD10 (as control, green) using small intestine and kidney tissues. In small intestine tissue, cubilin expression was observed with N-terminal antibody. Conversely, no expression was observed with C-terminal antibody. In kidney tissue, cubilin expression was observed with both N-terminal antibody (antibody 1) and C-terminal antibody (antibody 2). Kidney biopsy tissue from Neph224 with bilateral truncating variants showed no expression of cubilin with either antibody. Scale bar: 100 μm. (c) Western blot analysis for cubilin in small intestine and kidney tissue lysate using N-terminal antibody (antibody 1). β-actin was used as a loading control. In small intestine tissue lysates, a single band of 150–250 kD was detected. In kidney tissue lysates, a full-length band and a smaller band of 150–250 kD were detected. (d) Automated capillary western blot images of cubilin in small intestine and kidney tissue lysates using N-terminal antibody (antibody 1). A band corresponding to full-length cubilin (approximately 460 kD) was observed only in the kidney lysate, whereas a band of approximately 190 kD was observed in both kidney and small intestine lysate.

In the immunofluorescence of kidney and small intestine tissues with a cubilin antibody against the N-terminal region before CUB5–8 (antibody 1), protein expression was observed in both the kidney and small intestine. In contrast, with the cubilin antibody against the C-terminal region posterior to CUB5–8 (antibody 2), protein expression was observed only in the kidney, and no or very slight expression was observed in the small intestine. In addition, kidney biopsy tissues from Neph224 with bilateral truncating variants showed little or weak expression of cubilin with either antibody 1 or antibody 2. CD10, which is expressed in the brush border of the proximal tubules and the small intestine, was highly expressed in all tissues (Figure 3b).

When cubilin protein expression was examined by western blotting using an antibody against the N-terminus of cubilin (antibody 1), a band corresponding to full-length cubilin (approximately 460 kD) was observed only in the kidney lysate, whereas a band of approximately 190 kD was observed in both kidney and small intestine lysates (Figure 3c and d).

## Discussion

We present the clinical and pathological features of patients with proteinuria with *CUBN* variants and demonstrate that, among the 40 *CUBN* variants identified, certain hotspot variants are specific to the Japanese population. In addition to these clinical and genetic findings, we describe the molecular mechanisms that explain the genotype–phenotype correlation between IGS and chronic benign proteinuria.

Most of the variants were located behind the CUB8 domain, which is consistent with the results of previous studies.^10^^,^^14^^,^^21^ In our cohort, there were 11 variants detected in multiple families, and p.Cys1764Tyr and p.Ser1936Asn were detected in the largest number of 13 families. Because these variants were novel disease–related variants and were either absent or infrequent in the gnomAD and Japanese-specific single nucleotide polymorphism databases, they were considered Japanese-specific hotspot variants. In previous studies in Europe, the most common *CUBN* variant identified in families with isolated proteinuria was p.Tyr3018Ser,^10^^,^^14^ but this variant was not found in our cohort. This suggests that individuals of different ethnicities have different hotspots.

The clinical presentation of our patients with biallelic *CUBN* variants was similar to that reported previously.^10^^,^^14^ All patients were incidentally diagnosed with subclinical proteinuria and had no hypoalbuminemia, kidney dysfunction, or response to RAS inhibitors. Our data show no significant change in the absolute value of urinary β_2_-microglobulin. Proteinuria owing to *CUBN* variants is “tubular,” not “glomerular.” Distinguishing patients with proteinuria and *CUBN* variants from those with other inherited glomerular diseases (such as monogenic podocytopathies) through urinalysis may be challenging because urine β_2_-microglobulin is within a normal range. Dent disease, one of the most common types of monogenic tubular proteinuria, is characterized by trafficking defects in megalin and cubilin, resulting in impaired protein endocytosis.^22^ This disease shows a marked elevation of urine β_2_-microglobulin,^23^ which is different from *CUBN* abnormality, even in the same tubular proteinuria. These findings illustrate that β_2_-microglobulin reabsorption is dependent only on megalin, not cubilin.^24^^,^^25^

In Japan, all 3-year-old children undergo urinary screening, which is why most patients with *CUBN* variants were detected to have proteinuria at 3 years of age. Our findings demonstrate that proteinuria occurs during early childhood in patients with *CUBN* variants; in other words, if proteinuria is absent in infancy and appears later, chronic benign proteinuria is probably not suspected.

This study enabled a comprehensive investigation of kidney pathology associated with *CUBN* abnormalities. On light microscopy, although almost all patients showed minor glomerular abnormalities, 2 patients showed mesangial proliferative nephritis, and 1 patient showed FSGS. In addition, patients with *CUBN* variants often exhibited slight pathological changes. In electron microscopic findings, approximately half of the patients showed mild foot process effacement, and approximately one-third of the patients showed a thin basement membrane. These pathological findings resembled early changes seen in podocytopathies such as FSGS or Alport syndrome, which may lead to diagnostic difficulty, particularly in children, in whom histologic alterations at early disease stages often remain subtle. Several previous reports have described biallelic *CUBN* variants presenting with FSGS ± foot process effacement,^14^^,^^26^^,^^27^ and additional cases showing Alport-like abnormalities of the glomerular basement membrane.^28^

The reasons why podocyte changes occur in chronic benign proteinuria—a disorder primarily involving the proximal tubule—and whether these changes are primary or secondary remain unclear. Consistent with previous reports, patients in our cohort who presented with FSGS carried ≥ 1 truncating variant, suggesting that some glomerular changes may be primary rather than purely secondary. Although previous studies suggested that cubilin could be detected in rat and human glomeruli and in cultured human podocytes,^29^^,^^30^ these findings were not validated using knockout models and may instead reflect cubilin shed from proximal tubules.^31^ Furthermore, currently available human and mouse single-cell RNA-seq datasets demonstrate strong *CUBN* expression in proximal tubular cells but negligible expression in podocytes.^32^^,^^33^ Given that these patients occasionally exhibit glomerular alterations, and in light of recent evidence that thin-basement-membrane nephropathy—once regarded as “benign familial hematuria”—may actually belong to the Alport spectrum and carry a risk of chronic kidney disease progression,^34^^,^^35^ it seems premature to consider *CUBN*-related proteinuria entirely benign. Long-term observation will be essential to determine whether *CUBN*-related proteinuria remains truly benign throughout life.

We discovered an alternative *CUBN* transcript variant, in which a cryptic exon created within intron 28 resulted in a premature stop codon. According to the results of immunofluorescence and western blotting, an approximately 190-kD protein, which is thought to be the cubilin isoform lacking the C-terminus, is expressed in the small intestine and kidney, and can be produced by this novel transcript variant. In addition, our findings indicate that this novel transcript escapes nonsense-mediated decay and is translated into a stable protein. In patients with C-terminal *CUBN* variants, the isoform expressed in the small intestine that lacks the C-terminal region is not affected by these variants. Consequently, vitamin B12 malabsorption does not occur. Although the truncated intestinal isoform accounts for the preservation of vitamin B12 absorption in patients with C-terminal variants, the renal phenotype is more likely attributable to dysfunction of the full-length cubilin protein in the proximal tubule. In particular, C-terminal missense variants are considered to impair the structure or function of the full-length protein independently of the truncated isoform.

Although our data demonstrated the presence of the truncated *CUBN* transcript and its corresponding protein isoform in the intestine, we were unable to quantitatively compare the abundance of the truncated and full-length transcripts in intestinal and kidney tissues. Therefore, we cannot exclude the possibility that the full-length transcript is also expressed at low levels in the intestine. Previous animal studies have shown that cubilin contributes not only to vitamin B12 absorption but also to intestinal protein uptake, including albumin^36^^,^^37^; suggesting that full-length *CUBN* transcripts may well be expressed in the intestine as well.

This novel transcript variant is consistent with the genotype-phenotype correlation of previously reported patients. Referring to the latest Human Gene Mutation Database (HGMD), none of the variants posterior to exon 29 arise associated with megaloblastic anemia or IGS. In contrast, most of the variants before exon 28 were associated with megaloblastic anemia; however, all remaining variants associated with proteinuria had paired-allele variants posterior to exon 29. Further, the presence of intestinal transcript truncated directly after CUB8 in the Genotype-Tissue Expression database^10^ and the fact that vitamin B12 uptake is maintained if cubilin is truncated after CUB8^38^ are also consistent with the novel transcript in this study.

In Iso-Seq using human small intestine ileum and human kidney total RNA, some novel transcript variants were observed in addition to the transcript variant containing cryptic exon within intron 28. Notably, the transcript variant in which exon 25 is elongated on the 3' side without splicing intron 25 containing open reading frame was observed in large quantities (Supplementary Figure S1). In addition, we cloned this transcript variant using the 3'-RACE method. However, with reference to HGMD, 5 variants between exons 26 and 28 are associated with megaloblastic anemia, and it is likely that the transcript containing cryptic exon within intron 28 generates the functional isoform protein, rather than the transcript in which exon 25 is elongated. However, it has not been confirmed from which novel transcripts the isoform is produced, which is a limitation of the study.

Furthermore, several issues remain unresolved. First, the albumin binding site in cubilin has not yet been fully elucidated. One previous study using rat cubilin showed that CUB5–8 contains the intrinsic factor vitamin B12 and albumin-binding sites.^39^ However, based on the present findings and results by Bedin *et al.*,^10^ there is a strong possibility that the C-terminus of cubilin is important for the reabsorption of albumin. Another limitation of this study is that functional analyses of the truncated cubilin protein have not yet been fully conducted. Further studies are required to evaluate the stability of the truncated protein, its interaction with megalin, and its ability to mediate vitamin B12 and albumin uptake. Second, therapeutic modalities for kidney disease targeting cubilin have not yet been developed. Downregulating tubular reabsorption may be an effective strategy for the treatment of tubular injury, and cubilin could be a potential therapeutic target for kidney disease.^10^^,^^40^^,^^41^ In rats, targeting cubilin with antisense RNA has shown promising results in the amelioration of adriamycin-induced tubulointerstitial injury. This treatment led to decreased serum creatinine levels, improved glomerular filtration rate, and attenuated histological changes, despite an increase in albuminuria.^42^

In summary, PROCHOB is a tubulopathy that at first glance appears to be a podocytopathy and is currently diagnosed only by genetic analysis. However, the fact that the disease is nonresponsive to RAS inhibitors and that proteinuria is observed from early childhood is a sign that enables the diagnosis of this disease. Furthermore, our discovery of a novel *CUBN* transcript variant that explains the phenotypic differences between IGS and chronic benign proteinuria may elucidate the pathophysiology and establish new strategies to prevent tubular injury by targeting cubilin.

## Disclosure

KN is a member of the advisory groups Kyowa Kirin Co., Ltd., Toa Eiyo Ltd., Zenyaku Kogyo Co., Ltd., and Taisho Pharmaceutical Co., Ltd.; has a patent for developing exon skipping therapy for patients with Alport syndrome; received lecture fees from Ono Pharmaceutical Co., Ltd., Astellas Pharma Inc., Novo Nordisk Pharmaceuticals Ltd., Alexion Pharmaceuticals, Inc., Sumitomo Pharma Co., Ltd., Sanofi S.A. Otsuka Pharmaceutical Co., Ltd., Daiichi Sankyo Company Limited, and Miyarisan Pharmaceutical Co., Ltd.; received speaker bureaus from Sumitomo Pharma Co., Ltd., Chugai Pharmaceutical Co., Ltd., JCR Pharmaceuticals Co., Ltd., Sanofi S.A., Zenyaku Kogyo Co., Ltd., and Kyowa Kirin Co., Ltd.; and received a grant from Toa Eiyo Ltd, Zenyaku Kogyo Co., Ltd. and Torii Pharmaceutical Co., Ltd. TH received research funding from Otsuka Pharmaceutical Co., Ltd., Sysmex Corporation, and Zenyaku Kogyo Co., Ltd. All the other authors declared no competing interests.

## Supplementary Material

Supplementary File (PDF) Figure S1. Isoform sequencing (Iso-Seq) on human kidney and intestine. Figure S2. Minigene assay demonstrating aberrant splicing of *CUBN* variants. Figure S3. Electron microscopic findings in patients with biallelic *CUBN* variants. Figure S4. Electrophoresis and sequencing results of the novel CUBN transcript variant. Table S1. List of genes responsible for inherited kidney diseases screened by targeted sequencing. Table S2. Antibodies used in this study. Table S3. Details of CUBN variants detected in this study. Table S4. Segregation information of CUBN variants. Checklist (STREGA).
