# Supplementary material for: Clinical and Genetic Insights Into Isolated Proteinuria With CUBN Variants
Source: Kidney Int Rep. 2025 Dec 29;11(3):103754. doi: 10.1016/j.ekir.2025.103754 (PMC12857354; doi:10.1016/j.ekir.2025.103754)
Supplement: Supplementary File (PDF) — Figure S1. Isoform sequencing (Iso-Seq) on human kidney and intestine. Figure S2. Minigene assay demonstrating aberrant splicing of CUBN variants. Figure S3. Electron microscopic findings in patients with biallelic CUBN variants. Figure S4. Electrophoresis and sequencing results of the novel CUBN transcript variant. Table S1. List of genes responsible for inherited kidney diseases screened by targeted sequencing. Table S2. Antibodies used in this study. Table S3. Details of CUBN variants detected in this study. Table S4. Segregation information of CUBN variants. Checklist (STREGA). [file mmc1.pdf]

Supplementary materials

Title: Clinical and Genetic Insights into Isolated Proteinuria with *CUBN* Variants

Supplementary Figure S1.

a. Human Small Intestine

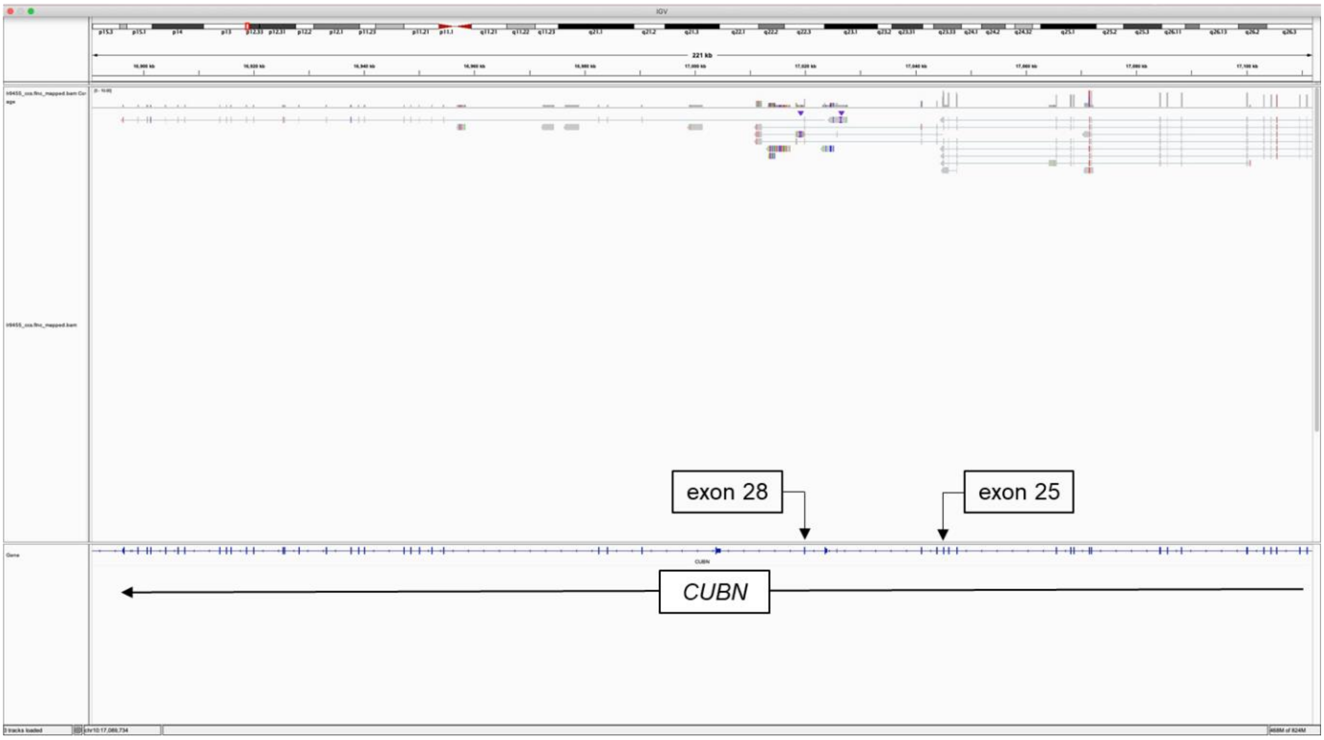

b. Human Kidney

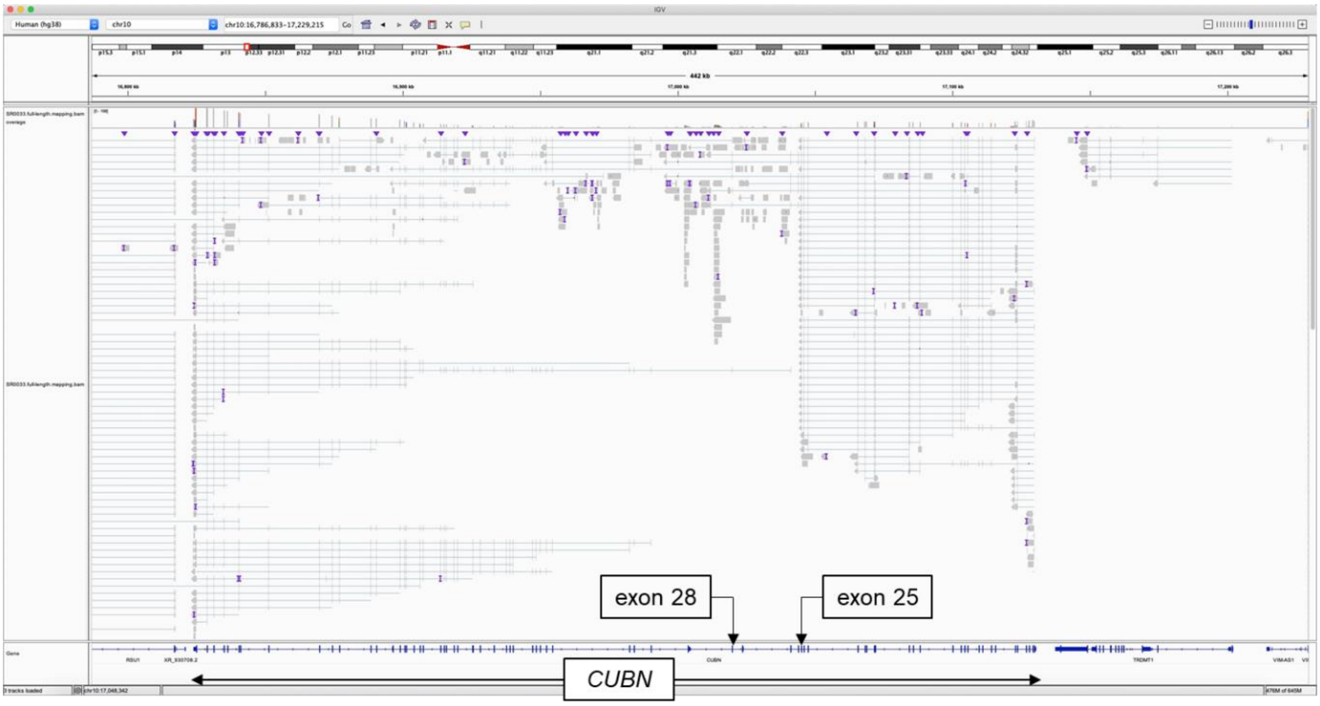

Supplementary Figure S1. Isoform sequencing (iso-seq) on human kidney total RNA and human ileum total RNA.

Iso-seq is the method of long-read RNA-sequencing that enables the identification of transcriptomes. Library preparation with human kidney total RNA (Takara Bio, Kusatsu, Japan) and human small intestinal ileum total RNA (BioChain, Newark, CA) was performed using the IsoSeq Express Oligo Kit and SMRTbell Express Template Prep Kit 2.0 (Pacific Biosciences, Menlo Park, CA). The constructed libraries were sequenced using a PacBio Sequel II with Sequel II Sequencing Kit 2.0 (Pacific Biosciences, Menlo Park, CA). All procedures were performed according to the manufacturer's instructions. To generate full-length isoforms, the IsoSeq workflow (https://github.com/PacificBiosciences/IsoSeq) was performed in the following four steps: consensus reads (ccs in SMRTLink v10.1.0), lima (lima in SMRTLink v10.1.0), refine (isoseq3 v3.3.0 with '--requier-polya --min-polya-length 20' parameter), and cluster (isoseq3 v3.3.0 with '--use-qvs' parameter). To identify novel isoforms, full-length isoforms were aligned to the Ensembl GRCh38 using minimap2 (v 2.19) with the '-x splice -uf -C 5 –secondary=no' parameters and visualized in IGV. a. Iso-seq of total RNA from the human small intestine, mainly consisting of sequences prior to exon 28, whereas sequences posterior to exon 28 are rarely observed. This iso-seq also contains several exon 25 elongated sequences. b. Iso-seq of total RNA from the human kidney. Sequences prior to exon 28 and sequences posterior to exon 28 were also observed. This iso-seq also contained exon 25 elongated sequences.

Supplementary Figure S2.

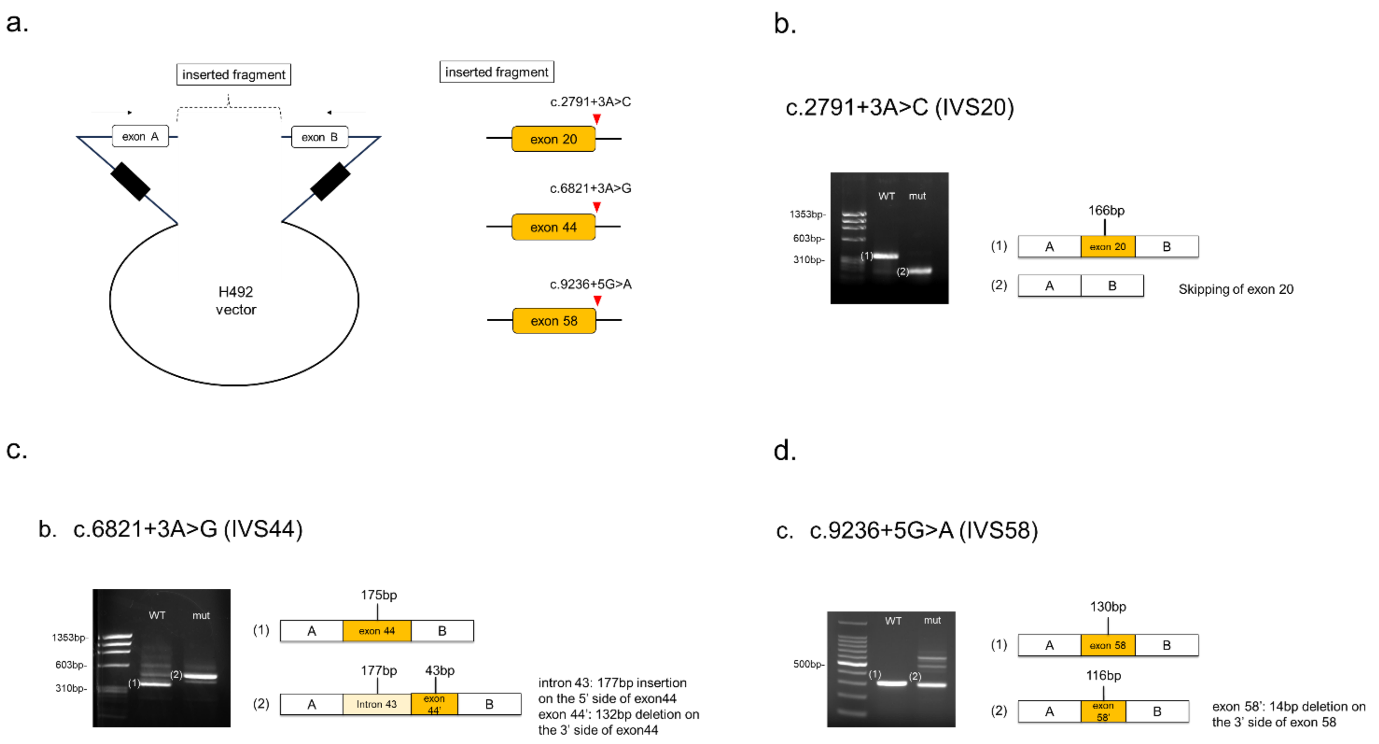

Supplementary Figure S2. Schematic representation of minigene constructs and RT-PCR-amplified products of *CUBN* splice variants. All variants exhibited aberrant splicing. a. Intron and exon boundaries of the variants inserted into the H492 vector between exons A and B. Variants in each inserted fragment are indicated by red arrows. b. RT-PCR amplification of the c.2791+3A>C hybrid minigene transcripts. Skipping of the whole exon 20 was also observed. c. RT-PCR amplified products of c.6821+3A>G hybrid minigene transcripts. Insertion on the 5’ side of exon 44 and deletion on the 3’ side of exon 44 were observed. d. RT-PCR amplified products of the c.9236+5G>A hybrid minigene transcript. Deletion on the 3’ side of exon 58 was observed. WT: wild type, mut: mutant

Supplementary Figure S3.

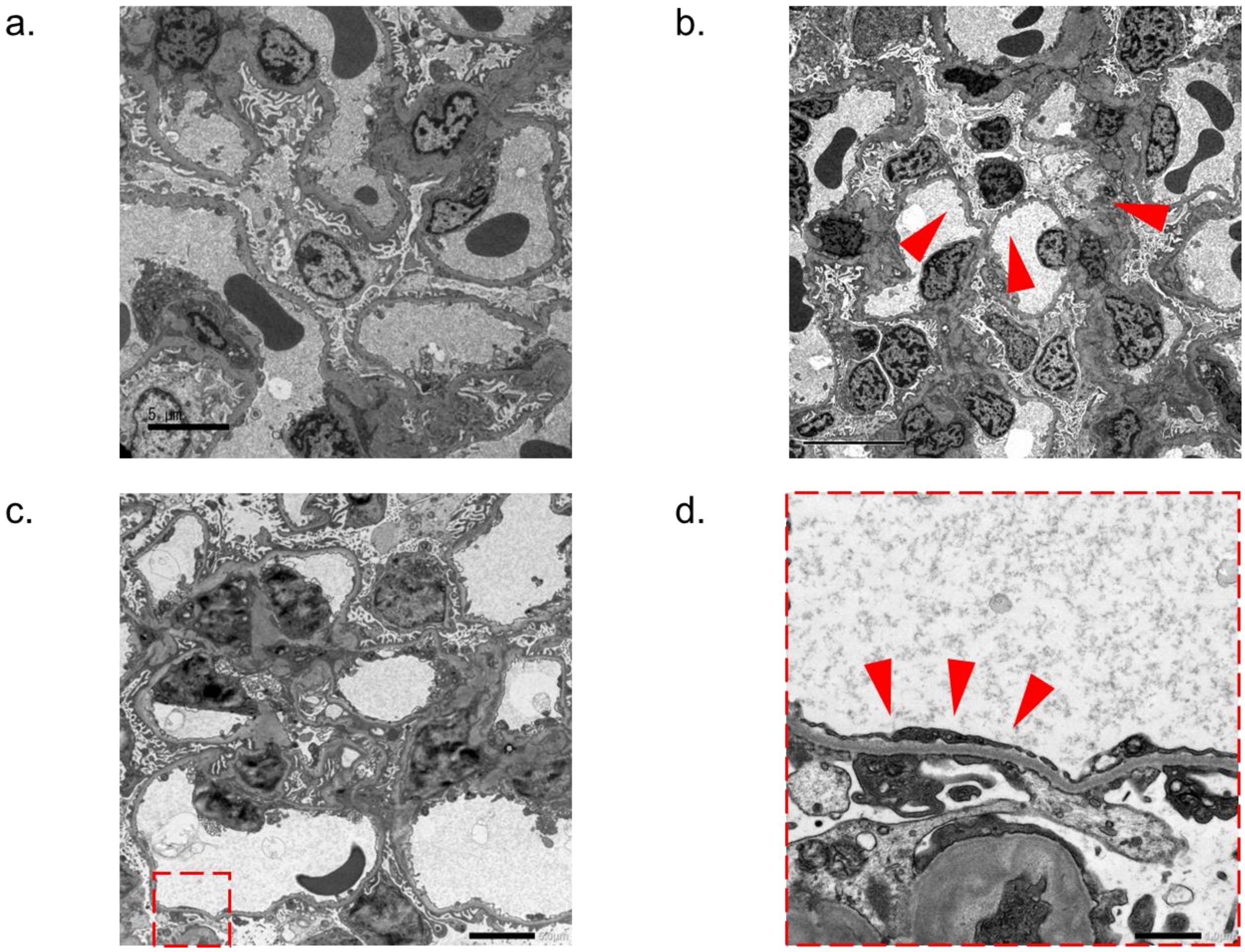

Supplementary Figure S3. Electron microscopic images of the patients with biallelic *CUBN* variants. a. Electron microscopy image of Neph255. No remarkable abnormalities were observed. Scale bar: 5 µm. b. Electron microscopy image of Neph558 brother. Mesangial-cell proliferation was also observed. Scale bar: 10 µm. c. Electron microscopy image of Neph558. Mesangial proliferation and partially thin basement membranes were also observed. Scale bar: 10 µm. d. Electron microscopy image of Neph558 (enlarged image of the red dotted line in c). Scale bar: 1 µm.

Supplementary Figure S4.

a.

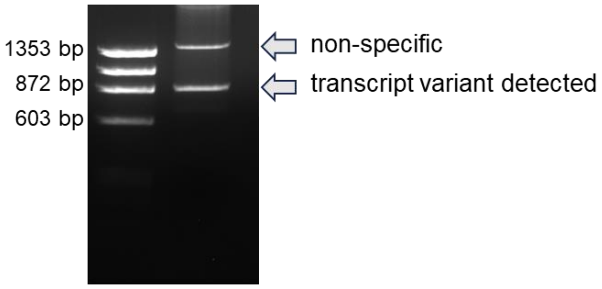

b.

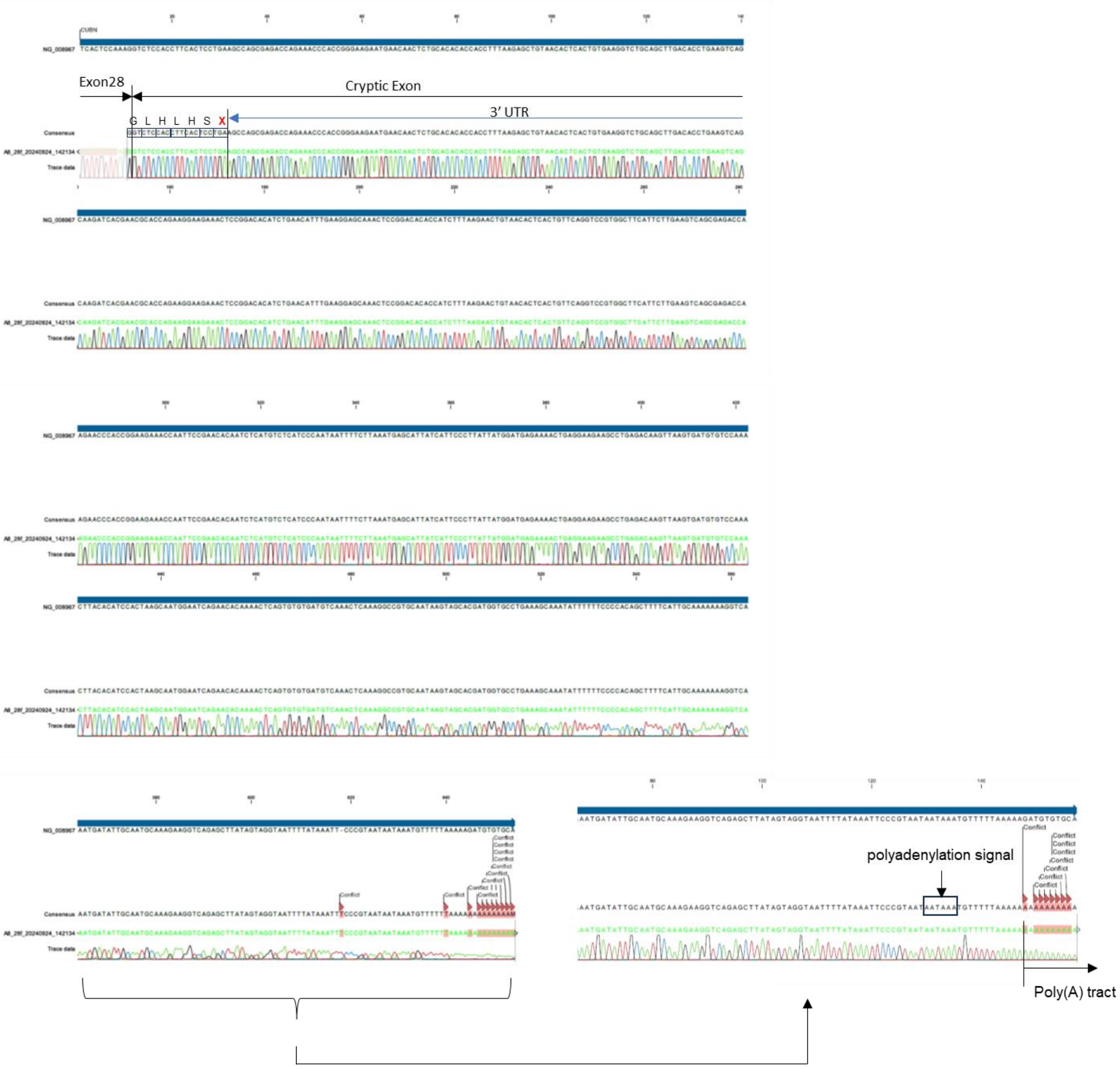

Supplementary Figure S4. Electrophoresis and sequencing images of novel *CUBN* transcript variant cloned by 3' RACE using human small intestine cDNA. a. Electrophoresis image. The left lane contains DNA size markers, and the right lane contains *CUBN* transcript variant. Each arrow indicates a novel transcript variant and a nonspecific product. b. Sequencing image. The cryptic exon beginning with g.17,001,178 (c.4169-10663) in intron 28 was generated posterior to exon 28, and the poly(A) tract was added after the polyadenylation signal (AAUAAA) in g.17,000,563-g.17,000,558. This novel transcript variant contains an open reading frame (ORF).

Supplementary Table S1. List of the genes responsible for inherited kidney diseases screened by targeted sequencing

SLC12A1 NM_000338.2	C3 NM_000064.3	AQP2 NM_000486.5	ARHGDIA NM_001185077.2	MAGI2 NM_012301.3
KCNJ1 NM_000220.4	CFB NM_001710.5	AVPR2 NM_000054.4	ADCK4 NM_024876.3	AVIL NM_ 006576.3
CLCNKB NM_000085.4	DGKE NM_003647.2	AVP NM_000490.4	TTC21B NM_024753.4	TNS2 NM_015319.2
BSND NM_057176.2	THBD NM_000361.2	COL4A3 NM_000091.4	NUP93 NM_014669.4	DLC1 NM_182643.2
CLCNKA NM_004070.3	CFHR1 NM_002113.2	COL4A4 NM_000092.4	NUP107 NM_020401.2	CDK20 NM_001039803.2
SLC12A3 NM_000339.2	ADAMTS13 NM_139025.4	COL4A5 NM_000495.4	NUP205 NM_015135.2	ITSN1 NM_001001132.1
CASR NM_000388.3	FN1 NM_212482.1	P3H2 NM_018192.4	NUP85 NM_024844.4	ITSN2 NM_147152.2
MAGED2 NM_177433.2	SLC4A1 NM_000342.3	CD151 NM_004357.5	NUP133 NM_018230.2	KIRREL1 NM_018240.6
CLDN10 NM_006984.4	ATP6V0A4 NM_020632.2	GLA NM_000169.2	NUP160 NM_015231.1	SGPL1 NM_003901.3
CFTR NM_000492.3	ATP6V1B1 NM_001692.3	UMOD NM_003361.3	CRB2 NM_173689.6	LMNA NM_170707.3
CLCN5 NM_000084.4	SLC4A4 NM_003759.3	MUC1 NM_002456.5	CUBN NM_001081.3	LAMA5 NM_005560.4
OCRL NM_000276.3	CA2 NM_000067.2	SEC61A1 NM_013336.3	EMP2 NM_001424.4	GAPVD1 NM_ 015635.3
EHD1 NM_001282444.2	EHHADH NM_001966.3	REN NM_000537.3	FAT1 NM_005245.3	ANKFY1 NM_ 001257999.2
SLC26A3 NM_000111.2	SLC34A1 NM_003052.4	EYA1 NM_000503.5	KANK1 NM_015158.3	GON7 NM_032490.4
KCNJ10 NM_002241.4	SLC2A2 NM_000340.1	SIX2 NM_016932.4	KANK2 NM_001136191.2	LAGE3 NM_006014.4
CLDN16 NM_006580.3	BCS1L NM_001257343	CD2AP NM_012120.2	KANK4 NM_181712.4	OSGEP NM_017807.3
CLDN19 NM_148960.2	GATM NM_001482.2	NPHS1 NM_004646.3	PDSS2 NM_020381.3	TPRKB NM_016058.3
FXYD2 NM_001680.4	HNF4A NM_175914.4	NPHS2 NM_014625.3	PTPRO NM_030667.2	TP53RK NM_033550.3
EGF NM_001963.4	NDUFAF6 NM_152416.3	PLCE1（NPHS3）NM_016341.3	XPO5 NM_020750.2	WDR4 NM_18669.5
TRPM6 NM_017662.4	CTNS NM_004937.2	SMARCAL1 NM_014140.3	ACTN4 NM_004924.4	WDR73 NM_032856.3
KCNA1 NM_000217.2	NR3C2 NM_000901.4	LAMB2 NM_002292.3	ANLN NM_018685.4	PRDM15 NM_001040424.3
CNNM2 NM_017649.4	SCNN1A NM_001038.5	SCARB2 NM_005506.3	ARHGAP24 NM_001025616.2	TRIM8 NM_030912.2
HNF1B NM_000458.3	SCNN1B NM_000336.2	COQ2 NM_015697.7	INF2 NM_022489.3	PODXL NM_005397.3
PCBD1 NM_000281.3	SCNN1G NM_001039.3	COQ6 NM_182476.2	LMX1B NM_002316.3	TBC1D8B NM_017752.2
ANK3 NM_020987.5	KLHL3 NM_017415.2	ITGA3 NM_002204.2	MYH9 NM_002473.5	MAFB NM_005461.5
CFH NM_000186.3	CUL3 NM_003590.4	ITGB4 NM_001005731.1	PAX2 NM_003987.3	
CFI NM_000204.3	WNK1 NM_018979.3	GLEPP1 (PTPRO) NM_030667.2	TRPC6 NM_004621.5	
MCP (CD46) NM_002389.4	WNK4 NM_032387.4	MYO1E NM_004998.3	WT1 NM_24426.5	

Supplementary Table S2. Antibodies used in this study

Antibody	Product name	Source	Identifier	Host	Dilution
**Immunofluorescence**					
CD10	Human/Mouse Neprilysin/CD10 Antibody	R&D systems	#200103	Rat	1:20
Cubilin N-terminal	cubilin (H-3)	Santa cruz	sc-518059	Mouse	1:50
Cubilin C-terminal	Anti-Cubilin antibody [EPR16259-27]-C-terminal	abcom	ab191073	Rabbit	1:2000
Secondary Antibody	Goat anti-Rat IgG (H+L) Cross-Adsorbed Secondary Antibody, Alexa Fluor™ 488	Invitrogen	A11006	-	1:100
Secondary Antibody	Goat anti-Rabbit IgG (H+L) Highly Cross-Adsorbed Secondary Antibody, Alexa Fluor™ 546	Invitrogen	A11035	-	1:100
Secondary Antibody	Goat anti-Mouse IgG (H+L) Highly Cross-Adsorbed Secondary Antibody, Alexa Fluor™ 546	Invitrogen	A11030	-	1:100
**Western Blot**					
Cubilin N-terminal	cubilin (H-3)	Santa cruz	sc-518059	Mouse	1:100
β-actin	β-Actin Antibody	Cell Signaling Technology	#4967	Rabbit	1:2000
Secondary Antibody	Anti-mouse IgG, HRP-linked Antibody	Cell Signaling Technology	#7076	-	1:2000
Secondary Antibody	Anti-mouse IgG, HRP-linked Antibody	Cell Signaling Technology	#7074	-	1:2000

Supplementary Table S3. The details of the variants detected in this study

Variants	Transcript	Exon	Families, n	dbSNP ID	gnomAD 4.1	Tommo54 K	*In silico* algorithm predictions for missense variant					ACMG	Reference
							REVEL	CADD	SIFT	Polyphen-2	Mutation taster		
c.796G>A	p.Glu266Lys	8	1	rs1161400848	3.72E-06	0.000018	0.887	24.5	Deleterious	Probably damaging	Benign	PM1+PM2+PM3+PP3 Likely Pathogenic	-
c.1399_1402del TGTC	p.Cys467Argfs*14	12	1	-	Absent	Absent	-	-	-	-	-	PVS1+PM2 Pathogenic	-
c.2791+3A>C	-	IVS20	1	-	Absent	Absent	-	-	-	-	-	PVS1+PM2 Pathogenic	-
c.3685_3686del CC	p.Pro1229Lysfs*2	26	1	-	-	-	-	-	-	-	-	PVS1+PM2+PM3 Pathogenic	-
c.4214C>A	p.Pro1405His	29	1	-	Absent	Absent	0.442	26.8	Deleterious	Probably damaging	Deleterious	PM1+PM2+PM3+PP3 Likely Pathogenic	-
c.4837C>T	p.Arg1613*	32	1	rs769881615	1.05E-05	0.000101	-	-	-	-	-	PVS1+PM2 Pathogenic	S1
c.4855+2C>G	-	32	3	rs772316719	4.10E-06	0.000295	-	-	-	-	-	PVS1+PM2+PP1 Pathogenic	S2
c.4907G>A	p.Arg1636Gln	33	1	rs538984401	6.84E-07	Absent	0.165	22	Tolerated	Probably damaging	Benign	PM1+PM2+PM3 Likely Pathogenic	-
c.5066A>G	p.Asp1689Gly	32	1	rs759994039	9.92E-06	Absent	0.215	23.1	Deleterious	Possibly damaging	Benign	PM1+PM2+PM3+PP3 Likely Pathogenic	-
c.5291G>A	p.Cys1764Tyr	36	13	rs986680465	5.47E-06	0.000175	0.632	24.9	Deleterious	Probably damaging	Deleterious	PM1+PM2+PM3+PP1+PP3 Likely Pathogenic	S3

c.5302_5304delATC	p.Ile1768del	36	3	rs775161946	6.20E-06	0.000037	-	-	-	-	-	PM2+PM3+PM4+PP1 Likely Pathogenic	-
c.5373C>A	p.Cys1791*	29	1	-	-	Absent	-	-	-	-	-	PVS1+PM2 Pathogenic	-
c.5380G>T	p.Asp1794Tyr	37	1	-	Absent	Absent	0.504	27.2	Deleterious	Probably damaging	Deleterious	PM1+PM2+PM3+PP3 Likely Pathogenic	-
c.5519dupG	p.Thr1841Hisfs*14	37	1	-	Absent	Absent	-	-	-	-	-	PVS1+PM2 Pathogenic	-
c.5733+1G>T	-	IVS38	4	rs1842587266	5.48E-06	0.000129	-	-	-	-	-	PVS1+PM2+PP1 Pathogenic	19
c.5806_07delinsAA	p.Ser1936Asn	39	13	rs1554796668	Absent	Absent	-	-	Deleterious	Probably damaging	-	PM1+PM2+PM3+PP1 Likely Pathogenic	-
c.5842G>A	p.Asp1948Asn	39	2	rs1285741474	7.44E-06	0.000009	0.524	24.4	Tolerated	Probably damaging	Benign	PM1+PM2+PM3 Likely Pathogenic	-
c.5854T>C	p.Ser1952Pro	39	1	-	Absent	Absent	0.131	22.9	Tolerated	Possibly damaging	Benign	PM1+PM2+PM3 Likely Pathogenic	-
c.5855C>T	p.Ser1952Leu	39	1	rs1842551920	0	Absent	0.087	22.8	Deleterious	Benign	Benign	PM1+PM2+PM3 Likely Pathogenic	-
c.6125-2A>G	-	IVS40	1	rs75386064	4.60E-04	Absent	-	-	-	-	-	PVS1 Likely Pathogenic	10
c.6821+3A>G	-	IVS44	5	rs767078847	1.61E-05	0.000203	-	-	-	-	-	PVS1+PM2+PP1 Pathogenic	S4
c.7010G>A	p.Gly2337Glu	46	1	-	Absent	0.000028	0.537	24.1	Deleterious	Probably damaging	Deleterious	PM1+PM2+PM3+PP3 Likely Pathogenic	-

c.7289T>C	p.Phe2430Ser	47	1	-	Absent	Absent	0.667	25.5	Deleterious	Possibly damaging	Deleterious	PM1+PM2+PM3+PP3 Likely Pathogenic	-
c.7352-3_7354del	-	48	4	rs1841809257	0	0.000018	-	-	-	-	-	PVS1+PM2+PP1 Pathogenic	20
c.7432T>C	p.Cys2478Arg	48	1	-	Absent	Absent	0.769	27.1	Deleterious	Probably damaging	Benign	PM1+PM2+PM3+PP3 Likely Pathogenic	-
c.7580_7581del	p.Cys2527*	49	1	-	3.19E-06	0.000028	-	-	-	-	-	PVS1+PM2 Pathogenic	-
c.7906C>T	p.Arg2636*	50	1	rs137998687	1.75E-04	Absent	-	-	-	-	-	PVS1+PM2+PM3+PP5 Pathogenic	27
c.8080A>G	p.Ile2694Val	52	1	-	Absent	Absent	0.074	17.81	Tolerated	Benign	Benign	PM1+PM2+PM3+BP4 Uncertain Significance	-
c.8748delT	p.Phe2916Leufs*29	55	1	-	Absent	Absent	-	-	-	-	-	PVS1+PM2 Pathogenic	-
c.9236+5G>A	-	IVS58	1	rs1458087960	8.05E-06	0.000013	-	-	-	-	-	PVS1+PM2 Pathogenic	-
c.9389T>C	p.Leu3130Pro	59	2	-	6.84E-06	Absent	0.525	25.1	Deleterious	Possibly damaging	Deleterious	PM1+PM2+PM3+PP3 Likely Pathogenic	-
c.9619G>T	p.Glu3207*	60	1	-	Absent	Absent	-	-	-	-	-	PVS1+PM2 Pathogenic	-
c.9994T>C	p.Cys3332Arg	62	1	-	2.74E-06	Absent	0.550	24.6	Deleterious	Probably damaging	Deleterious	PM1+PM2+PP3 Uncertain Significance	-
c.10245C>A	p.Tyr3415*	64	3	rs147730705	2.48E-06	0.000203	-	-	-	-	-	PVS1+PM2+PP1+PP5 Pathogenic	19

c.10342T>G	p.Cys3448Gly	64	1	-	Absent	Absent	0.635	25	Tolerated	Probably damaging	Deleterious	PM1+PM2+PM3+PP3 Likely Pathogenic	-
c.10535G>A	p.Gly3512Asp	66	1	-	Absent	Absent	0.536	25.1	Deleterious	Probably damaging	Deleterious	PM1+PM2+PM3+PP3 Likely Pathogenic	-
c.10562C>G	p.Ser3521*	66	2	-	Absent	Absent	-	-	-	-	-	PVS1+PM2 Pathogenic	-
c.10764+1G>A	-	IVS66	1	rs374982220	5.45E-05	0.000009	-	-	-	-	-	PVS1+PM2 Pathogenic	S5
incl.ex. 67 & ex. 1, 2 of RSU1			1	-	Absent	Absent	-	-	-	-	-	PVS1+PM2 Pathogenic	-
incl.ex. 64, 65, 66, 67 & ex. 1, 2 of RSU1			1	-	Absent	Absent	-	-	-	-	-	PVS1+PM2+PM3 Pathogenic	-

Supplementary Table S4. Segregation information of *CUBN* variants

No.	Patient ID	Variants	Transcript	Exon	Segregation F	Segregation M
1	A729	c.10342T>G	p.Cys3448Gly	64	-	+
		c.10562C>G	p.Ser3521*	66	+	-
2	Neph17	c.5806_07delinsAA	p.Ser1936ASN	39	-	+
		c.9619G>T	p.Glu3207*	60	+	-
3	Neph195	c.5291G>A	p.Cys1764Tyr	36	+	-
		c.5806_07delinsAA	p.Ser1936ASN	39	-	+
4	Neph224	c.5733+1G>T	-	IVS38	-	+
		c.10245C>A	p.Tyr3415*	64	+	-
5	Neph255	c.5291G>A	p.Cys1764Tyr	36	-	+
		c.5842G>A	p.Asp1948Asn	39	+	-
6	Neph278	c.7352-3_7354del	-	48	-	+
		c.10764+1G>A	-	IVS66	+	-
7	Neph358	c.5291G>A	p.Cys1764Tyr	36	ND	ND
		c.5733+1G>T	Exon skipping	IVS38	ND	ND
8	Neph359	c.2791+3A>C	-	IVS20	+	-
		c.9389T>C	p.Leu3130Pro	59	-	+
9	Neph423	c.4855+2C>G	p.Ile1590Met	32	ND	ND
		c.9994T>C	p.Cys3332Arg	62	ND	ND
10	Neph496	c.5733+1G>T	-	IVS38	ND	+
		c.5855C>T	p.Ser1952Leu	39	ND	-
11	Neph515	c.5291G>A	p.Cys1764Tyr	36	+	-
		c.5806_07delinsAA	p.Ser1936ASN	39	-	+
12	Neph527	c.7352-3_7354del	-	48	+	-
		c.5806_07delinsAA	p.Ser1936ASN	39	-	+
13	Neph558	c.7580_7581del	p.Cys2527*	49	-	+
		c.6821+3A>G	-	IVS44	+	-
14	Neph576	c.4855+2C>G	p.Ile1590Met	32	ND	+
		c.7352-3_7354del	-	48	ND	-
15	Neph580	c.5291G>A	p.Cys1764Tyr	36	+	-
		c.5854T>C	p.Ser1952Pro	39	-	+
16	Neph603	c.5291G>A	p.Cys1764Tyr	36	MD	-
		c.4855+2C>G	p.Ile1590Met	32	ND	+
17	Neph606	c.4837C>T	p.Arg1613*	32	+	-

		c.5066A>G	p.Asp1689Gly	32	-	+
18	Neph624	c.4214C>A	p.Pro1405His	29	ND	-
		c.5373C>A	p.Cys1791*	29	ND	+
19	Neph627	c.5291G>A	p.Cys1764Tyr	36	+	-
		c.7010G>A	p.Gly2337Glu	46	-	+
20	Neph635	c.8748delT	p.Phe2916Leufs*29	55	+	-
		c.6821+3A>G	-	IVS44	-	+
21	Neph642	c.8080A>G	p.Ile2694Val	52	-	+
		c.9236+5G>A	-	IVS58	+	-
22	Neph655	c.5291G>A	p.Cys1764Tyr	36	ND	ND
		c.5806_07delinsAA	p.Ser1936Asn	39	ND	ND
23	Neph658	c.5806_07delinsAA	p.Ser1936Asn	39	ND	+
		c.7289T>C	p.Phe2430Ser	47	ND	-
24	Neph668	c.4907G>A	p.Arg1636Gln	33	+	-
		c.5806_07delinsAA	p.Ser1936Asn	39	-	+
25	Neph697	c.5519dupG	p.Thr1841Hisfs*14	37	-	+
		c.6821+3A>G	-	IVS44	+	-
26	Neph708	c.1399_1402delTGTC	p.Cys467Argfs*14	12	-	+
		c.9389T>C	p.Leu3130Pro	59	+	-
27	Neph711	c.5291G>A	p.Cys1764Tyr	36	-	+
		c.5842G>A	p.Asp1948Asn	39	+	-
28	Neph716	c.7432T>C	p.Cys2478Arg	48	-	+
		c.10562C>G	p.Ser3521*	66	+	-
29	Neph719	c.5291G>A	p.Cys1764Tyr	36	+	-
		c.5806_07delinsAA	p.Ser1936Asn	39	-	+
30	Neph732	c.5806_07delinsAA	p.Ser1936Asn	39	-	+
		c.5380G>T	p.Asp1794Tyr	37	+	-
31	Neph749	c.5302_5304delATC	p.Ile1768del	36	+	-
		c.10535G>A	p.Gly3512Asp	66	-	+
32	Neph761	c.5806_07delinsAA	p.Ser1936Asn	39	ND	ND
		c.7352-3_7354del	-	48	ND	ND
33	Neph766	c.5291G>A	p.Cys1764Tyr	36	ND	-
		c.5806_5807delinsAA	p.Ser1936Asn	39	ND	+
34	Neph771	c.4855+2C>G	p.Ile1590Met	32	ND	+
		c.5302_5304delATC	p.Ile1768del	36	ND	-

35	Neph781	c.5733+1G>T	-	IVS38	-	+
		c.10245C>A	p.Tyr3415*	64	+	-
36	Neph800	c.3685_3686delCC	p.Pro1229Lysfs*2	26	ND	ND
		c.6125-2A>G	-	IVS40	ND	ND
37	Neph811	c.796G>A	p.Glu266Lys	8	-	+
		gross deletion	-	-	ND	ND
38	Neph819	c.5806_5807delinsAA	p.Ser1936Asn	39	+	-
		c.10245C>A	p.Tyr3415*	64	-	+
39	Neph815	c.6821+3A>G	-	IVS44	ND	ND
		gross deletion	-	-	ND	ND
40	Neph845	c.5291G>A	p.Cys1764Tyr	36	-	+
		c.5302_5304del	p.Ile1768del	36	+	-
41	Neph860	c.5291G>A	p.Cys1764Tyr	36	ND	ND
		c.5806_5807delinsAA	p.Ser1936Asn	39	ND	ND
42	Neph865	c.6821+3A>G	-	IVS44	+	-
		c.7906C>T	p.Arg2636*	50	-	+

Reporting checklist for genetic association study.

Based on the STREGA guidelines.

Instructions to authors

Complete this checklist by entering the page numbers from your manuscript where readers will find each of the items listed below.

Your article may not currently address all the items on the checklist. Please modify your text to include the missing information. If you are certain that an item does not apply, please write "n/a" and provide a short explanation.

Upload your completed checklist as an extra file when you submit to a journal.

In your methods section, say that you used the STREGAreporting guidelines, and cite them as:

Little J, Higgins JP, Ioannidis JP, Moher D, Gagnon F, von Elm E, Khoury MJ, Cohen B, Davey-Smith G, Grimshaw J, Scheet P, Gwinn M, Williamson RE, Zou GY, Hutchings K, Johnson CY, Tait V, Wiens M, Golding J, van Duijn C, McLaughlin J, Paterson A, Wells G, Fortier I, Freedman M, Zecevic M, King R, Infante-Rivard C, Stewart A, Birkett N; STrengthening the REporting of Genetic Association Studies. STrengthening the REporting of Genetic Association Studies (STREGA): An Extension of the STROBE Statement.

		Reporting Item	Page Number
**Title and abstract**			
Title	#1a	Indicate the study's design with a commonly used term in the title or the abstract	p.1,2

Abstract	#1b	Provide in the abstract an informative and balanced summary of what was done and what was found	p.2
**Background/rationale**			
	#2	Explain the scientific background and rationale for the investigation being reported	p.3-4
**Objectives**			
	#3	State specific objectives, including any prespecified hypotheses. State if the study is the first report of a genetic association, a replication effort, or both.	p.4
**Study design**			
	#4	Present key elements of study design early in the paper	p.4-5
**Setting**			
	#5	Describe the setting, locations, and relevant dates, including periods of recruitment, exposure, follow-up, and data collection	p.4-5
**Eligibility criteria**			

	#6a	Cohort study – Give the eligibility criteria, and the sources and methods of selection of participants. Describe methods of follow-up. Case-control study – Give the eligibility criteria, and the sources and methods of case ascertainment and control selection. Give the rationale for the choice of cases and controls. Cross-sectional study – Give the eligibility criteria, and the sources and methods of selection of participants. Give information on the criteria and methods for selection of subsets of participants from a larger study, when relevant.	not applicable
	#6b	Cohort study – For matched studies, give matching criteria and number of exposed and unexposed. Case-control study – For matched studies, give matching criteria and the number of controls per case.	not applicable
**Variables**			
	#7a	Clearly define all outcomes, exposures, predictors, potential	not applicable

		confounders, and effect modifiers. Give diagnostic criteria, if applicable	
	#7b	Clearly define genetic exposures (genetic variants) using a widely-used nomenclature system. Identify variables likely to be associated with population stratification (confounding by ethnic origin).	not applicable
**Data sources/measurement**			
	#8a	For each variable of interest give sources of data and details of methods of assessment (measurement). Describe comparability of assessment methods if there is more than one group. Give information separately for for exposed and unexposed groups if applicable.	p.4-5
	#8b	Describe laboratory methods, including source and storage of DNA, genotyping methods and platforms (including the allele calling algorithm used, and its version), error rates and call	p.5

		rates. State the laboratory / centre where genotyping was done. Describe comparability of laboratory methods if there is more than one group. Specify whether genotypes were assigned using all of the data from the study simultaneously or in smaller batches.	
**Bias**			
	#9a	Describe any efforts to address potential sources of bias	not applicable
	#9b	Describe any efforts to address potential sources of bias	not applicable
**Study size**			
	#10	Explain how the study size was arrived at	not applicable
**Quantitative variables**			
	#11	Explain how quantitative variables were handled in the analyses. If applicable, describe which groupings were chosen, and why. If applicable, describe how effects of treatment were dealt with.	not applicable
**Statistical methods**			

#12a	Describe all statistical methods, including those used to control for confounding. State software version used and options (or settings) chosen.	p.4
#12b	Describe any methods used to examine subgroups and interactions	not applicable
#12c	Explain how missing data were addressed	not applicable
#12d	If applicable, explain how loss to follow-up was addressed	not applicable
#12e	Describe any sensitivity analyses	not applicable
#12f	State whether Hardy-Weinberg equilibrium was considered and, if so, how.	not applicable
#12g	Describe any methods used for inferring genotypes or haplotypes	not applicable
#12h	Describe any methods used to assess or address population stratification.	not applicable
#12i	Describe any methods used to address multiple comparisons or to control risk of false positive findings.	not applicable

	#12j	Describe any methods used to address and correct for relatedness among subjects	not applicable
**Participants**			
	#13a	Report numbers of individuals at each stage of study—eg numbers potentially eligible, examined for eligibility, confirmed eligible, included in the study, completing follow-up, and analysed. Give information separately for for exposed and unexposed groups if applicable. Report numbers of individuals in whom genotyping was attempted and numbers of individuals in whom genotyping was successful.	not applicable
	#13b	Give reasons for non-participation at each stage	not applicable
	#13c	Consider use of a flow diagram	not applicable
**Descriptive data**			
	#14a	Give characteristics of study participants (eg demographic, clinical, social) and information on exposures and potential confounders. Give information separately for exposed and	not applicable

		unexposed groups if applicable. Consider giving information by genotype	
	#14b	Indicate number of participants with missing data for each variable of interest	Supplementary materials
	#14c	Cohort study – Summarize follow-up time, e.g. average and total amount.	not applicable
**Outcome data**			
	#15	Cohort study Report numbers of outcome events or summary measures over time.Give information separately for exposed and unexposed groups if applicable. Report outcomes (phenotypes) for each genotype category over time Case-control study – Report numbers in each exposure category, or summary measures of exposure.Give information separately for cases and controls . Report numbers in each genotype category. Cross-sectional study – Report numbers of outcome events or summary measures. Give	not applicable

		information separately for exposed and unexposed groups if applicable. Report outcomes (phenotypes) for each genotype category	
**Main results**			
	#16a	Give unadjusted estimates and, if applicable, confounder-adjusted estimates and their precision (eg, 95% confidence interval). Make clear which confounders were adjusted for and why they were included	not applicable
	#16b	Report category boundaries when continuous variables were categorized	not applicable
	#16c	If relevant, consider translating estimates of relative risk into absolute risk for a meaningful time period	not applicable
	#16d	Report results of any adjustments for multiple comparisons	not applicable
**Other analyses**			
	#17a	Report other analyses done—e.g., analyses of subgroups and interactions, and sensitivity analyses	not applicable

	#17b	Report other analyses done—e.g., analyses of subgroups and interactions, and sensitivity analyses	not applicable
	#17c	Report other analyses done—e.g., analyses of subgroups and interactions, and sensitivity analyses	not applicable
**Key results**			
	#18	Summarise key results with reference to study objectives	p.10
**Limitations**			
	#19	Discuss limitations of the study, taking into account sources of potential bias or imprecision. Discuss both direction and magnitude of any potential bias.	p.13
**Interpretation**			
	#20	Give a cautious overall interpretation considering objectives, limitations, multiplicity of analyses, results from similar studies, and other relevant evidence.	p.12-14
**Generalisability**			

	#21	Discuss the generalisability (external validity) of the study results	not applicable
**Funding**			
	#22	Give the source of funding and the role of the funders for the present study and, if applicable, for the original study on which the present article is based	p.15

None The STREGA checklist is distributed under the terms of the Creative Commons Attribution License CC-BY. This checklist can be completed online using https://www.goodreports.org/, a tool made by the EQUATOR Network in collaboration with Penelope.ai

Supplementary References

S1. Wang F, Zhang Y, Mao J, et al. Spectrum of mutations in Chinese children with steroid-resistant nephrotic syndrome. *Pediatr Nephrol*. 2017;32:1181-1192.

S2. Jung J, Lee JH, Seo GH, et al. Genetic diagnosis of kidney disease by whole exome sequencing and its clinical application. *Clin Genet*. 2023;104:298-312.

S3. Takata A, Nakashima M, Saitsu H, et al. Comprehensive analysis of coding variants highlights genetic complexity in developmental and epileptic encephalopathy. *Nat Commun*. 2019;10:2506.

S4. Rao J, Liu X, Mao J, et al. Genetic spectrum of renal disease for 1001 Chinese children based on a multicenter registration system. *Clin Genet*. 2019;96:402-410.

S5. Yang H, He L, Gong H, et al. Identification of novel pathogenic variants of CUBN in patients with isolated proteinuria. *Mol Genet Genomic Med*. 2024;12:e2353.
